# Comparative efficacy of psychological interventions for internet gaming disorder: a meta-analysis of randomized controlled trials

**DOI:** 10.3389/fpsyt.2025.1619138

**Published:** 2025-07-10

**Authors:** Ruofei Wang, Yueqian Zhang, Zikang Liu, Hanwen Dong, Zhao Zhang, Kebing Yang, Yan Liu, Rongjiang Zhao, Qingyan Yang, Yajuan Niu

**Affiliations:** ^1^ Department of Psychology, Chengde Medical University, Chengde, China; ^2^ Beijing Huilongguan Hospital, Beijing, China

**Keywords:** psychological intervention, internet gaming disorder, moderating effect, rct, meta-analysis

## Abstract

**Background:**

This study employed a systematic review and meta-analysis to evaluate the efficacy of standalone psychological interventions for internet gaming disorder in randomized controlled trials. It further compared the effectiveness of psychological interventions across domestic and international studies, various intervention modalities, and different intervention durations. The goal is to provide empirical support for optimizing intervention strategies.

**Methods:**

A computerized search was conducted to identify randomized controlled trials on psychological interventions for gaming addiction published between 2015 and 2025 in EMBASE, the Cochrane Library, PubMed, CNKI, VIP, and Wanfang databases. Two researchers independently assessed the quality of the included studies and extracted relevant data. Meta-analysis was conducted using Review Manager 5.4 and Comprehensive Meta-Analysis Version 3. Subgroup analyses were performed with region, intervention modality, and intervention duration as moderator variables. A random-effects model was used to estimate the overall effect size. Heterogeneity among studies and publication bias were assessed.

**Results:**

In total, six studies were included, involving 372 participants. The results of the meta-analysis indicate that psychological interventions can alleviate symptoms of internet gaming disorder (SMD = -0.06, 95%CI: -0.99 to -0.20, P = 0.003). Subgroup analyses revealed that domestic psychological interventions (SMD = -0.89, 95%CI:-1.25 to -0.52, P < 0.00001), offline interventions (SMD = -0.67, 95%CI: -1.17 to -0.17, P = 0.008), and short-term interventions (SMD = -0.82, 95%CI: -1.27 to -0.37, P = 0.0004) were more effective in treating internet gaming disorder.

**Conclusions:**

Psychological interventions demonstrated significant therapeutic effects on internet gaming disorder; Specifically, domestic psychological interventions, offline interventions, and short-term interventions demonstrated superior efficacy compared to foreign interventions, online interventions, and long-term interventions, respectively. However, due to the limited number of included studies and substantial heterogeneity, meta-regression was not conducted; instead, subgroup analyses were employed to explore potential sources of heterogeneity.

**Systematic Review Registration:**

https://www.crd.york.ac.uk/prospero/, identifier CRD420251023957.

## 1 Introduction

In 2018, the World Health Organization officially classified internet gaming disorder (IGD) as a mental and behavioral disorder in the 11th edition of the International Classification of Diseases (ICD-11) (1). Internet gaming disorder is defined as a pattern of gaming behavior characterized by impaired control over gaming, persistent or recurrent gaming activity, increasing priority given to gaming over other interests and daily activities, and the continuation or escalation of gaming despite significant impairment in social functioning (2, 3). Although the Diagnostic and Statistical Manual of Mental Disorders, Fifth Edition, Text Revision (DSM-5-TR) has not yet included internet gaming disorder as an official diagnostic category, it has recognized it as a condition warranting further study. The primary diagnostic criteria continue to follow the nine core symptoms outlined in the Diagnostic and Statistical Manual of Mental Disorders, Fifth Edition (DSM-5): preoccupation with gaming, withdrawal symptoms, increased tolerance, unsuccessful attempts to reduce gaming, abandonment of other activities, continued gaming despite known problems, deceiving others about gaming time, using gaming to alleviate negative emotions, and significant impairment in functioning (4, 5). The DSM-5-TR encourages empirical research on internet gaming disorder from multiple dimensions, including behavioral manifestations, neurobiological mechanisms, comorbid psychiatric disorders, and cross-cultural differences, which is crucial for determining its clinical significance, pathological mechanisms, and effective intervention strategies (4, 6).

In recent years, epidemiological studies on internet gaming disorder have indicated that the condition is particularly prevalent among adolescents, with global prevalence rates varying across different countries and cultural contexts (7). A systematic review reported that the global average prevalence of internet gaming disorder ranges from approximately 3.05% to 6.04%, with significant regional variations (8, 9). Specifically, the prevalence rates are 0.3% to 1.0% in the United States (10), approximately 10% in China (11), and 18.2% in Brazil (12). In certain countries, the prevalence is even higher; for example, in Saudi Arabia, the internet gaming disorder prevalence among females is 19% (13), and it reaches up to 21.85% among male adolescents (14). Moreover, a large body of research has demonstrated significant comorbidity between internet gaming disorder and various psychological disorders, including depression, anxiety, impulse control disorders, and attention-deficit/hyperactivity disorder, with depression and anxiety symptoms being particularly prominent (15, 16). Relevant surveys indicate that approximately 9.2% of adolescents with internet gaming disorder exhibit significant anxiety symptoms, 15.1% show signs of depression, and 10.9% experience difficulties with impulse control (2, 17). This suggests that the treatment of internet gaming disorder should incorporate targeted interventions that address its comorbid psychiatric and psychological conditions.

Psychological intervention refers to the application of psychological theories and methods, using structured or unstructured approaches to systematically influence and modify an individual's perceptions, cognition, emotions, and behaviors, in order to restore balance between the individual and their environment and achieve therapeutic goals (18). Psychological interventions encompass psychotherapy, counseling, and crisis intervention, and can be delivered through various modalities such as online or offline formats (online psychological intervention refers to remote psychological interventions, whereas offline psychological intervention denotes face-to-face interventions delivered in person), as well as short-term or long-term approaches (18). Psychological interventions, as an important means of treating addictive behaviors, have been widely applied in the treatment of substance addictions (such as alcohol, tobacco, and drugs) and behavioral addictions (such as gambling and internet addiction), accumulating a substantial body of empirical research (19, 20). Currently, cognitive behavioral therapy and motivational interviewing have become mainstream psychological interventions (21, 22). Cognitive behavioral therapy is based on Beck's cognitive theoretical model, which posits that individuals' perceptions and interpretations of events influence their emotions and behaviors (23). In the intervention for individuals with internet gaming disorder, cognitive behavioral therapy aims to help individuals identify negative automatic thoughts, explore and address core beliefs, thereby facilitating cognitive restructuring and improving their emotional states and behavioral patterns (24). Motivational interviewing is a collaborative, person-centered communication approach aimed at enhancing individuals' intrinsic motivation, emphasizing the client's autonomy and respecting their willingness to change. In interventions targeting internet gaming disorder, motivational interviewing helps individuals resolve ambivalence and elicit intrinsic motivation for behavioral change (25, 26). The intervention process is typically based on the OARS technique, which employs core strategies such as open-ended questions, affirmation, reflective listening, and summarization to build a supportive relationship and guide individuals toward healthier behavioral patterns (27). In addition, integrated psychological intervention models have also demonstrated certain efficacy in the treatment of internet gaming disorder. This model is typically grounded in evidence-based psychotherapeutic approaches such as cognitive behavioral therapy and motivational interviewing, and integrates various intervention techniques including relaxation training, play therapy, and group therapy to address individuals' cognition, emotions, and behaviors from multiple dimensions, thereby enhancing the systematic nature and effectiveness of treatment (28).

In the field of addiction intervention, traditional face-to-face psychological interventions are considered to be more effective due to their strong interactivity and personalized characteristics. In recent years, with the advancement of internet technology, online psychological interventions have gradually emerged, demonstrating a certain degree of convenience and practicality (29). However, there remains considerable controversy regarding comparative studies on the effectiveness of online versus offline psychological interventions. Some studies have also indicated that the effectiveness of psychological interventions for internet gaming disorder varies across different cultural contexts (30). Meanwhile, variations in intervention duration also affect the stability of intervention outcomes (31). Therefore, there is some controversy regarding the optimal intervention duration for treating internet gaming disorder.

Based on the above background, this study proposes the following hypotheses: Psychological intervention has a significant therapeutic effect on internet gaming disorder (H_1_); Domestic psychological interventions are more effective than those abroad (H_2_); Offline interventions are more effective than online interventions (H_3_); A shorter intervention duration is more effective than a longer intervention duration (H_4_). To test these hypotheses, this study conducts a systematic review and meta-analysis, synthesizing data from RCT studies in China and other countries, comparing the overall therapeutic effects of psychological interventions on internet gaming disorder, and exploring the moderating effects of cultural background, intervention methods, and intervention duration on intervention outcomes.

## 2 Materials and methods

### 2.1 Study registration

We conducted and reported this systematic review according to the PRISMA statement (32). This study was preregistered on the PROSPERO platform under the registration number CRD420251023957.

### 2.2 Inclusion criteria

The PICO framework emphasizes the elements of Population(P), Intervention (I), Comparison (C), and Outcome (O). The following content is presented according to the four elements of PICO.

#### 2.2.1 Types of studies

All studies were published randomized controlled trials (RCTs), limited to Chinese or English language, using psychological interventions to treat internet gaming disorder.

#### 2.2.2 Type of participants

All participants included in the studies were adolescents or college students aged 12 years or older, regardless of gender, and their assessment scores met the minimum criteria for addictive behaviors on the relevant scales. Participants did not have concomitant severe physical illnesses, intellectual disabilities, or organic psychiatric disorders. All participants provided informed consent from their legal guardians.

#### 2.2.3 Type of interventions and comparisons

The intervention group primarily received psychological interventions, including cognitive behavioral therapy, motivational interviewing, comprehensive psychological interventions (CBT, relaxation training, game therapy), and RAMBSP online courses, while the control group received conventional treatment or placebo.

#### 2.2.4 Outcome indicators

The primary outcome measure of this study was the change in symptoms of internet gaming disorder. To ensure comprehensive and scientific assessment, the included original studies employed multiple validated scales to evaluate internet gaming disorder, including the IGDS9-SF, IAT, CIAS-G, BSMAS, and GDAS.

### 2.3 Exclusion criteria

1)The study type is not clearly stated. 2)The study participants, study type, intervention measures, and outcome measures do not meet the inclusion criteria. 3)Review articles, commentaries, meta-analyses, conference abstracts, research plans, etc. 4)Studies for which the full text cannot be accessed, effective data cannot be extracted, or original data could not be obtained. 5)Studies that are published repeatedly.

### 2.4 Literature search strategy

Computer-based searches were conducted in EMBASE, PubMed, Cochrane Library, CNKI, VIP, and Wanfang databases, with a search period from 2015 to 2025. Both Chinese and English searches used a combination of subject headings, free terms, and Boolean logic operators. English search terms: internet addiction, Internet Addiction Disorder, Psychosocial Intervention, randomized controlled trial, and corresponding free-text terms in each database. Chinese search terms: "Gaming addiction(游戏成瘾)", "Gaming disorder(游戏障碍)", "Online gaming addiction(网络游戏成瘾)", "Psychological intervention(心理干预)", "Psychotherapy(心理治疗)", "RCT(随机对照)". (The detailed search terms are provided in **Supplementary Material Appendix A**).

### 2.5 Literature screening and data extraction

The studies were selected based on the PRISMA guidance (32). Two researchers independently screened the literature based on the inclusion and exclusion criteria and, after reviewing the full texts of the preliminarily included studies, conducted a second screening; any discrepancies were resolved through discussion (33). The consistency of literature screening between the two researchers was assessed using the Kappa coefficient, which was 1, indicating perfect agreement. The data extraction primarily included the following elements: author, publication year, country, study type, sample characteristics (sample size, age), intervention methods, control methods, and outcome measures.

### 2.6 Evaluation of literature quality

According to evidence-based medicine guidelines, the Cochrane systematic review "Risk of Bias" assessment tool was employed to evaluate the quality of the included studies across six domains: random sequence generation, allocation concealment, blinding, completeness of outcome data, selective reporting of results, and other sources of bias (34). During the statistical analysis, quality assessments were classified as follows: five or more items indicated a low risk of bias; three to four items indicated a moderate risk of bias; and fewer than three items indicated a high risk of bias.

### 2.7 Statistical methods

This study used RevMan Manager 5.4 and Comprehensive Meta-Analysis V3 (CMA) to conduct the meta-analysis. The standardized mean difference was adopted as the effect size measure (35). The confidence interval was 95%. Tests revealed heterogeneity among the studies; therefore, a random-effects model was employed. Sensitivity analysis was conducted using the leave-one-out method, bias was assessed via Egger's and Begg's tests, and subgroup analyses were performed to explore the sources of heterogeneity.

## 3 Results

### 3.1 Literature search results

**Figure 1:**
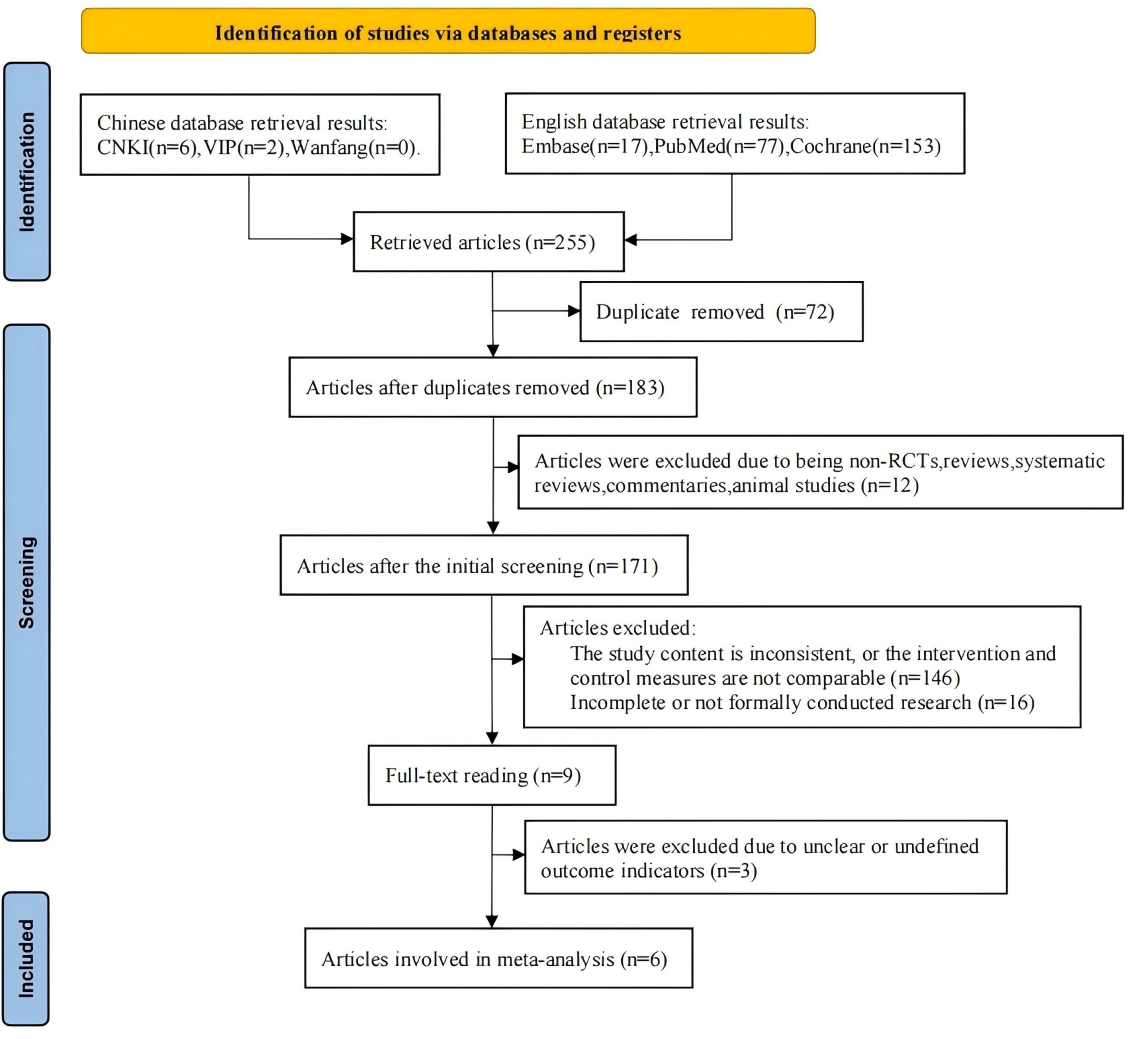
Literature search process.

The initial search retrieved 255 articles; after removing duplicates, 183 remained; excluding non-RCTs, reviews, systematic reviews, commentaries, animal studies, etc., 171 remained; screening abstracts to exclude those with mismatched study content or inconsistent intervention and control measures left 25; further excluding incomplete studies or those without formal research resulted in 9 articles. After full-text review and exclusion of studies with unclear outcome measures, 6 articles were ultimately included based on the predefined inclusion and exclusion criteria. See **Figure 1**.

### 3.2 Basic characteristics of the included studies

Of the six included studies, three were conducted in China and three abroad. Three studies implemented offline treatment, and three implemented online interventions. Four studies had intervention durations of ≤8 weeks, and two studies had durations >8 weeks. Two studies employed the Internet Addiction Test (IAT), one study used the Internet Gaming Disorder Scale–Short Form (IGDS9-SF), one study used the Chinese Internet Addiction Scale–Gaming version (CISA-G), one study used the Bergen Social Media Addiction Scale (BSMAS), and one study used the Gaming Disorder Assessment Scale (GDAS) to assess the severity of gaming addiction. The basic characteristics of the included studies are shown in **Table 1**.

**Table 1:** The basic characteristics of the research literature included in this study.

Author & year	*n*	Gender status	Age(M ± SD)	Intervention content	Frequency	Outcome indicators
Georgekutty et al., 2023 (36)	C=15	not reported	16-19	ACRIP online course	8 weeks, 8 sessions	IGDS9-SF
	E=15					
Jamal et al., 2023 (37)	C=30	C:male 20 female10	20.12 ± 1.06	group CBT	8 weeks, 8 sessions	IAT
	E=30	E:male 18 female12				
Yinan et al., 2023 (38)	C=51	C:male34 female5	16.36 ± 0.93	ICBT	8 weeks, 8 sessions	CIAS-G
	E=54	E:male34 female4				
Zeliha et al., 2023 (39)	C=38	C:male36 female18	C:21.8 ± 1.6	RAMBSP online course	12 weeks, 12 sessions	BSMAS
	E=39	E:male35 female16	E:21.5 ± 1.4			
Qing hai et al., 2017 (40)	C=20	not reported	C:14.08 ± 1.49	group psychological intervention	4 months, 17 sessions	IAT
	E=20		E:13.90 ± 1.37			
Wangzhe et al., 2019 (41)	C=30	C:male18 female12	C:14.87 ± 1.50	brief intervention method	15 minutes, 1 session	GDAS
	E=30	E:male16 female14	E:14.97 ± 1.10			

C represents the control group, E represents the experimental group; IGDS9-SF refers to the Internet Gaming Disorder Scale; IAT refers to the Internet Addiction Test; CIAS-G refers to the Chen Internet Addiction Scale-Gaming Version; BSMAS refers to the Bergen Social Media Addiction Scale; GDAS refers to the Game Disorder Assessment Scale; CBT refers to Cognitive Behavioral Therapy; ICBT refers to Integrated Cognitive Behavioral Therapy; ACRIP refers to the Acceptance and Cognitive Restructuring Intervention Program; RAMBSP refers to the Comprehensive Psychological Intervention Treatment.

### 3.3 Results of the quality assessment of the studies

**Figure 2:**
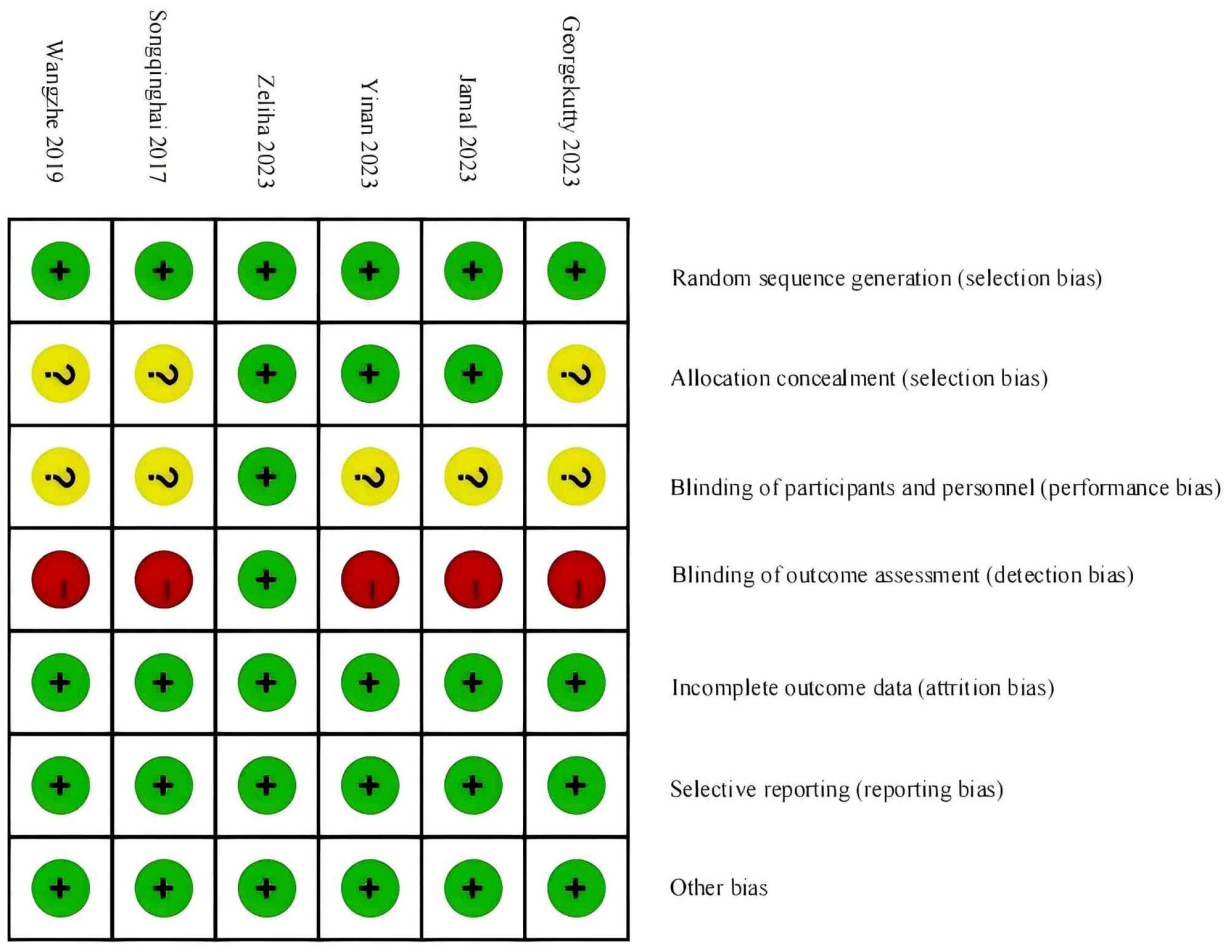
Schematic diagram of the methodological quality evaluation of literature.

**Figure 3:**
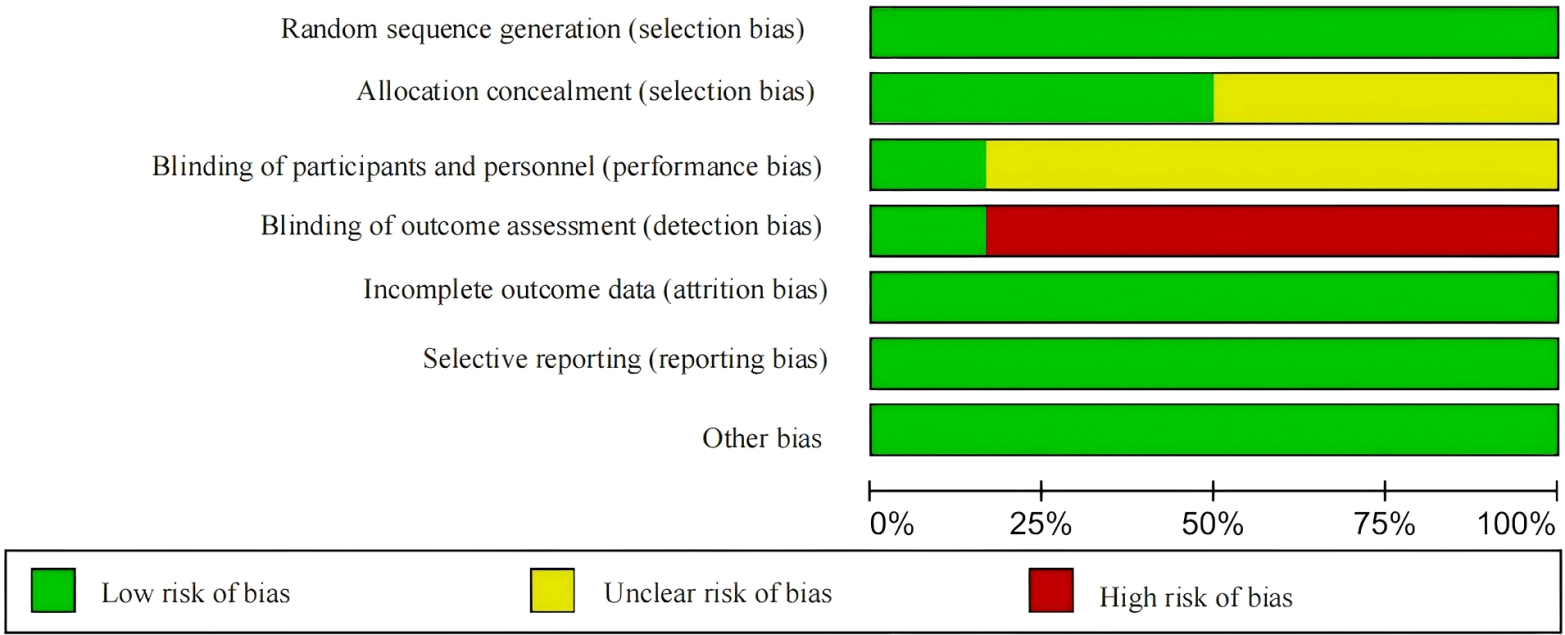
Proportional chart of the methodological quality assessment of the literature.

According to the Cochrane Risk of Bias Assessment Tool, the quality of the six included studies was evaluated. Among them, three studies were assessed as having a low risk of bias, and three studies were assessed as having a moderate risk of bias. See **Figure 2**. A statistical chart of the proportion of each item is shown in **Figure 3**.

### 3.4 Meta-analysis results

#### 3.4.1 Overall effect test

**Figure 4:**
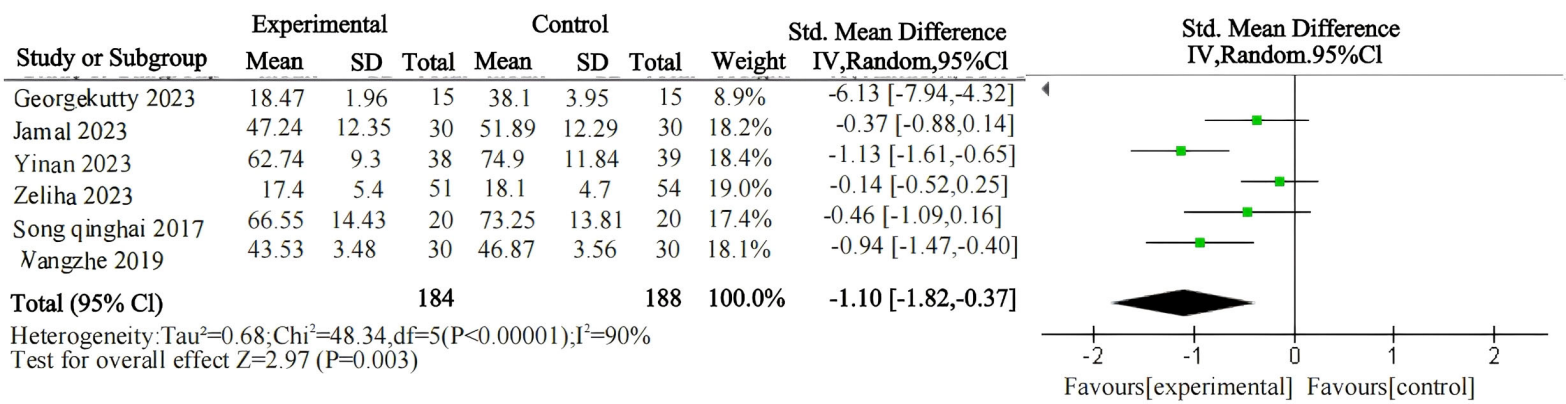
Forest plot of meta-analysis on the relationship between psychological intervention and internet gaming disorder (before sensitivity analysis).

**Figure 5:**
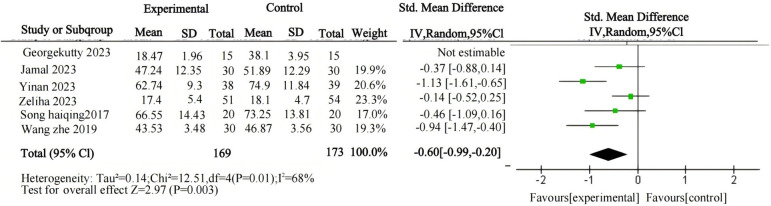
Forest plot of meta-analysis on the relationship between psychological intervention and internet gaming disorder (after sensitivity analysis).

First, an overall effect test was conducted on the entire sample, revealing that psychological interventions are effective in treating gaming addiction. A comprehensive heterogeneity test was conducted on the included studies (I² = 90%, P < 0.00001), indicating a high level of heterogeneity among the studies (**Table 2**; **Figure 4**). After conducting a sensitivity analysis and excluding one study, the heterogeneity test yielded revised results (I² = 68%, P < 0.00001), showing a substantial reduction in heterogeneity, which decreased to a moderate level (**Table 3**; **Figure 5**). Therefore, a random-effects model was adopted. The observed heterogeneity among multiple data sets in this meta-analysis suggests the potential presence of underlying moderator variables. On the other hand, the analysis of the negative sign preceding the value indicates the potential for treating gaming addiction. The pooled effect size of psychological interventions for treating gaming addiction is d = -0.6, indicating a significant therapeutic effect. According to Cohen's (1988) conventional interpretation, an effect size of 0.2 is considered a small effect, 0.20 < d < 0.80 represents a medium effect, and an effect size greater than 0.8 indicates a large effect. The results of the two-tailed test (P = 0.003) indicate that the pooled statistic for multiple data sets is statistically significant, with a 95% confidence interval of (-0.99, -0.20). The above data suggest that psychological interventions for gaming addiction have achieved favorable results, and the research hypothesis H_1_ is validated.

**Table 2:** Summary table of the relationship between psychological intervention and the effectiveness on internet gaming disorder (before sensitivity analysis).

Effect model	Independent sample	Homogeneity test			Two-tailed test		Effect size and 95% confidence interval
		X^2^	P	I^2^	Z	P	
random effects model	6	48.34	P<0.001	90%	2.97	P=0.003	-1.10 (-1.82,-0.37)

**Table 3:** Summary table of the relationship between psychological intervention and the effectiveness on internet gaming disorder (after sensitivity analysis).

Effect model	Independent sample	Homogeneity test			Two-tailed test		Effect size and 95% confidence interval
		X^2^	P	I^2^	Z	P	
random effects model	5	12.51	P=0.01	68%	2.97	P=0.003	-0.60 (-0.99,-0.20)

#### 3.4.2 Bias assessment

Due to the inclusion of fewer than 10 studies in this meta-analysis, CMA software was used to conduct Egger's and Begg's tests to assess bias. The Egger's test revealed an upper limit of 11.63 and a lower limit of -20.50, including 0, indicating no publication bias. The Begg's test revealed P > 0.05, indicating no publication bias.

#### 3.4.3 Moderation effect test

Based on the overall effect test, the possibility of underlying moderator variables was identified. Therefore, this study conducts hypothesis testing on the moderator variables affecting the relationship between psychological intervention and gaming addiction (**Table 4**). The results of the test were obtained through subgroup analysis of three moderator variables. The subgroup analysis results from both domestic and international studies show a high level of heterogeneity in effect size differences between the two groups (I² = 86.6%), indicating that domestic and international factors have a certain impact on the relationship between psychological intervention and gaming addiction. Specifically, the domestic group showed a larger effect size in treating gaming addiction (d = -0.89), indicating a large effect, while the effect size for the international group was smaller (d = -0.22), representing a medium effect. The effect size was higher in the domestic group compared to the international group. The subgroup analysis of intervention methods showed no heterogeneity in effect size differences between the two groups (I² = 0%), significantly reducing the heterogeneity of the overall effect, indicating that the type of intervention has a significant impact on the relationship between psychological intervention and gaming addiction. Specifically, offline interventions produced a larger effect size in treating gaming addiction (d = -0.67), indicating a medium effect, while online interventions had a smaller effect size (d = -0.51), also representing a medium effect. The effect size was higher for offline interventions compared to online interventions. The subgroup analysis of intervention duration showed moderate heterogeneity in effect size differences between the two groups (I² = 76.8%), indicating that the intervention duration has a certain impact on the relationship between psychological intervention and gaming addiction. Specifically, intervention durations of ≤8 weeks produced a larger effect size in treating gaming addiction (d = -0.82), indicating a large effect, while intervention durations of >8 weeks had a smaller effect size (d = -0.23), representing a medium effect. The effect size was higher for interventions lasting ≤8 weeks. The test results confirmed the research hypothesis H_2_、H_3_、H_4._

**Table 4:** Summary table of the moderation effect test in the meta-analysis of psychological intervention on internet gaming disorder effectiveness.

Moderator variable	Homogeneity test			Category	Effect size and 95% confidence interval	I^2^	Two-tailed test		Number of studies	Sample size
	X^2^	P	I^2^				Z	P		
Domestic and international	7.45	0.006	86.6%	Domestic	-0.89 (-1.25, -0.52)	26%	4.75	<0.001	3	177
				international	-0.22 (-0.53,0.08)	0%	1.42	0.16	2	165
Intervention methods	0.11	0.74	0%	Online	-0.51 (-0.31,0.27)	82%	1.29	0.02	2	165
				Offline	-0.67 (-1.17, -0.17)	61%	2.65	0.008	3	177
Intervention duration	4.31	0.04	76.8%	≤ 8 weeks	-0.82 (-1.27, -0.37)	57%	3.55	0.0004	3	197
				> 8 weeks	-0.23 (-0.55,0.10)	0%	1.35	0.18	2	145

## 4 Discussion

A total of six eligible studies were included in this research, and their methodological quality and risk of bias were systematically assessed. The results of the quality assessment indicated that these studies demonstrated high methodological stability and reliability. In the heterogeneity analysis, the I² value of the six studies reached 90%, indicating a high degree of heterogeneity among the studies. To further explore the sources of heterogeneity, a sensitivity analysis was conducted in this study. The results showed that after excluding one study, the I² value decreased to 68%, indicating a substantial reduction in heterogeneity. This suggests that the excluded study was likely the primary source of heterogeneity, and thus, five studies were ultimately included. The analysis revealed that the overall effect size of psychological interventions on internet gaming disorder reached a moderate level (d = -0.6) and was statistically significant, thereby confirming the research hypothesis that psychological interventions have a therapeutic effect on internet gaming disorder. This finding is consistent with previous studies (42).

The psychological interventions included in this study primarily consisted of cognitive behavioral therapy, motivational interviewing, and integrated intervention strategies combining cognitive behavioral therapy, relaxation training, and game therapy. The fact that three of the included studies utilized cognitive behavioral therapy indicates its high prevalence in current interventions for gaming disorder, which is closely related to the maturity of its theoretical foundation and the standardization of its implementation (43). Cognitive behavioral therapy grounded in cognitive and behavioral theories (23), emphasizes that maladaptive behaviors arise from irrational cognitions, with interventions focusing on cognitive restructuring and behavioral training to improve patients' thought patterns and behavioral responses (44), thereby alleviating addictive behaviors (45). Furthermore, some studies employed motivational interviewing based on humanistic psychology and self-determination theory (46), emphasizing behavior change through enhancing individuals' intrinsic motivation. Its person-centered approach facilitates the establishment of a positive therapeutic alliance during the early intervention phase, thereby creating favorable psychological conditions for subsequent addiction behavior correction (47). However, integrated psychological interventions combining multiple approaches demonstrate more significant therapeutic effects. Compared to single-modality interventions, these integrated methods typically merge diverse theoretical frameworks and techniques, such as combining CBT's structured cognitive restructuring with physiological regulation mechanisms from relaxation training and the emphasis on expression and connection in game therapy (48, 49). Such approaches can simultaneously operate across multiple dimensions, thereby enhancing the overall effectiveness of the intervention.

Among the six studies included in this research, one reported that all participants had comorbid depression and were treated with sertraline (Zoloft) at a dosage of 50 mg/day. However, the intervention group concurrently employed integrated intervention methods to improve internet gaming disorder. Another study measured symptoms of depression, anxiety, and stress using the Depression, Anxiety, and Stress Scale-21 items (DASS-21) alongside internet gaming disorder assessments. However, participants currently undergoing antidepressant treatment were excluded. The study aimed to reduce levels of depression, anxiety, and stress through CBT, thereby assisting participants in alleviating internet gaming disorder. However, the intervention studies included in this paper exhibit certain limitations regarding the consideration of relevant comorbidities. Although existing studies have demonstrated that internet gaming disorder is often significantly comorbid with psychological issues such as depression, anxiety, impulse control disorders, and attention-deficit/hyperactivity disorder (16, 50, 51). However, the other four studies included in this research neither systematically assessed nor intervened on these comorbid symptoms, and even treated them as exclusion criteria. While this approach enhanced the homogeneity of the intervention samples to some extent, it also limited the external validity of the study findings.

The overall heterogeneity of this study was 68%. When using the study location (domestic vs. international) as a moderator variable, the I² value of the combined subgroups was 86%, indicating that substantial heterogeneity remained within this grouping. However, further analysis revealed that the I² value for domestic studies was 26%, while that for international studies was 0%, indicating a high level of consistency among international studies, whereas some degree of heterogeneity remained among domestic studies. Therefore, the study source (domestic vs. international) may be one of the key factors contributing to the overall heterogeneity. Additionally, the results indicate that the effect size for the domestic group is higher than that for the international group. This suggests that, compared to international studies, domestic psychological interventions have a more significant therapeutic effect on internet gaming disorder. In China, influenced by confucian cultural traditions (52), society places a strong emphasis on family responsibility, and most adolescents are expected to obey parental authority. Parents often express concerns and resistance toward pharmacological treatments (53), showing a preference for non-pharmacological psychological interventions for their children. Meanwhile, Chinese adolescents are often reluctant to disclose their emotional states and gaming disorder issues due to social and self-stigmatization (54). Stigmatization not only exacerbates their psychological burden but may also lead to social rejection and labeling, thereby further reducing their willingness to accept psychiatric medication and diminishing their treatment motivation (55, 56). In contrast, psychotherapy is often perceived as a more acceptable form of intervention due to its greater emphasis on humanistic care and confidentiality (57). This form of intervention reduces stigmatization and safeguards personal privacy while providing a safe environment for clients, thereby enhancing their engagement in treatment and, to some extent, improving the actual effectiveness of psychotherapy (58). Moreover, psychological interventions in domestic practice have undergone localized adaptations, making them more attuned to the personality traits and developmental needs of local adolescents. This cultural adaptability further enhances the effectiveness of the intervention (59).

Furthermore, when intervention method was used as a moderator variable in the analysis, the I² value for the overall subgroup analysis was 0%. Further stratified analysis revealed that the I² value for the online intervention group was as high as 82%, while the I² value for the offline intervention group was 61%, both indicating a high degree of heterogeneity. This result suggests that variations in intervention modalities may be a significant source of overall heterogeneity. In particular, online psychological interventions may be influenced by several uncontrollable factors, such as platform instability, limited frequency of therapist-client interactions, and poor client adherence. These issues may collectively contribute to the high level of heterogeneity observed (60, 61). Analysis of the effect sizes for both groups revealed that the effect size for the offline intervention group was higher than that for the online intervention group. This suggests that, compared to online psychological interventions, offline psychological interventions have a more significant therapeutic effect in improving internet gaming disorder. This may be attributed to the face-to-face interaction between the therapist and the client in offline psychological interventions, which fosters greater personal engagement and facilitates the development of a stronger therapeutic alliance (62). Moreover, since individuals with Internet gaming disorder primarily engage in problematic behaviors online, offline psychological interventions may help disrupt their connection to these maladaptive online behavior patterns (63). In contrast, although online psychological interventions have advantages in accessibility and convenience, they may have certain limitations in establishing a therapeutic relationship, providing immediate feedback, and enhancing individual motivation (64–66). However, some studies have found that online psychological interventions have become a new trend in the treatment of patients with depression and anxiety, demonstrating certain efficacy and greater acceptability (67). However, in intervention studies targeting Internet gaming disorder, multiple studies have found that offline psychological interventions demonstrate more significant effects in alleviating the symptoms of the disorder (62, 68). The findings of this study also support this conclusion, indicating that offline interventions exhibit superior efficacy compared to online interventions in reducing addictive behaviors. Therefore, the results of this study further support the superiority of offline psychological interventions in the treatment of internet gaming disorder, while also suggesting that future research should explore how to optimize online intervention models to improve their effectiveness.

Finally, when intervention duration was used as a moderator variable in the analysis, the I² value for the overall subgroup analysis was 76.8%, indicating that the intervention duration may be one of the key factors influencing the consistency of the studies. Further analysis revealed that studies with an intervention duration of ≤8 weeks had an I² value of 57%, indicating moderate heterogeneity, while studies with an intervention duration of >8 weeks had an I² value of 0%, suggesting relatively consistent results in this group. This may be due to the inclusion of one study with a very short intervention duration of 15 minutes in the shorter intervention duration group, which is likely too brief. Analysis of the effect sizes for both groups revealed that the subgroup with an intervention duration of ≤8 weeks had a significantly higher effect size than the subgroup with an intervention duration of >8 weeks. This result suggests that, compared to longer-duration interventions, shorter-duration psychological interventions may have greater advantages in improving gaming addiction. Short-term interventions may be more effective in maintaining individuals' treatment motivation and reducing adherence decline due to excessively long intervention durations (69). Additionally, short-term interventions are typically more structured, providing high-intensity, targeted psychological support and behavioral adjustment strategies within a shorter time frame, leading to more significant intervention effects (70). However, long-term interventions may focus more on consolidating treatment effects, preventing relapse, and promoting deeper behavioral and cognitive changes, so their effects may gradually emerge in the later stages (71).

This study also has certain limitations. Firstly, due to certain uncontrollable factors, some relevant literature may not have been obtained or retrieved, resulting in a limited sample size included in the study, which precluded meta-regression analysis and thus affected the comprehensiveness and depth of the research. Secondly, although this study conducted bias assessment and sensitivity analyses which improved heterogeneity to some extent, the heterogeneity remained high, thereby limiting the generalizability of the findings. Therefore, future studies should further expand the search scope, include more relevant literature, and explore the efficacy of psychological interventions for gaming addiction more comprehensively.

## 5 Conclusions

In conclusion, this study systematically evaluated the efficacy of psychological interventions in the treatment of internet gaming disorder, and the results demonstrated that psychological interventions have favorable clinical effects in alleviating symptoms of internet gaming disorder. Further analysis of three moderating variables differences in psychological interventions between domestic and international contexts, intervention methods, and intervention duration revealed that the intervention effects were more pronounced in domestic studies compared to those conducted abroad. Offline interventions were more effective than online interventions; Interventions of shorter duration were more effective than those of longer duration. In summary, this study systematically confirmed the positive role of psychological interventions in the treatment of gaming addiction and provided a reference for optimizing intervention models. In future clinical practice, priority may be given to brief offline psychological intervention models, particularly integrated interventions based on cognitive-behavioral therapy within domestic educational settings. Meanwhile, intervention content can be tailored more specifically to participants with diverse cultural backgrounds and behavioral characteristics. Additionally, hybrid intervention models combining online and offline approaches can be explored to enhance therapeutic outcomes.

## Data Availability

The original contributions presented in the study are included in the article/**Supplementary Material**. Further inquiries can be directed to the corresponding author.
