# Supplementary material for: Comparative efficacy of psychological interventions for internet gaming disorder: a meta-analysis of randomized controlled trials
Source: Front Psychiatry. 2025 Jul 10;16:1619138. doi: 10.3389/fpsyt.2025.1619138 (PMC12288662; doi:10.3389/fpsyt.2025.1619138)
Supplement: Supplementary file 1 [file Table1.docx]

Appendix A

**Search syntax**

**Search syntax for English-language databases：**

**PUBMED**

Search: ((("Internet Addiction Disorder"[Mesh]) OR ((((((((((((((((((((((((((Addiction Disorders, Internet[Title/Abstract])) OR (Addiction Disorder, Internet[Title/Abstract])) OR (Disorder, Internet Addiction[Title/Abstract])) OR (Disorders, Internet Addiction[Title/Abstract])) OR (Internet Addiction Disorders[Title/Abstract])) OR (Internet Addiction[Title/Abstract])) OR (Addiction, Internet[Title/Abstract])) OR (Addictions, Internet[Title/Abstract])) OR (Internet Addictions[Title/Abstract])) OR (Internet Gaming Disorder[Title/Abstract])) OR (Disorder, Internet Gaming[Title/Abstract])) OR (Disorders, Internet Gaming[Title/Abstract])) OR (Gaming Disorder, Internet[Title/Abstract])) OR (Gaming Disorders, Internet[Title/Abstract])) OR (Internet Gaming Disorders[Title/Abstract])) OR (Smartphone Addiction[Title/Abstract])) OR (Addiction, Smartphone[Title/Abstract])) OR (Addictions, Smartphone[Title/Abstract])) OR (Smartphone Addictions[Title/Abstract])) OR (Social Media Addiction[Title/Abstract])) OR (Addiction, Social Media[Title/Abstract])) OR (Addictions, Social Media[Title/Abstract])) OR (Media Addiction, Social[Title/Abstract])) OR (Media Addictions, Social[Title/Abstract])) OR (Social Media Addictions[Title/Abstract]))) AND (("Psychosocial Intervention"[Mesh]) OR (((((((Intervention, Psychosocial) OR (Interventions, Psychosocial)) OR (Psychosocial Interventions)) OR (Psychological Intervention)) OR (Intervention, Psychological)) OR (Interventions, Psychological)) OR (Psychological Interventions)))) AND (randomized controlled trial[Publication Type] OR randomized[Title/Abstract] OR placebo[Title/Abstract]) Filters: from 2015 - 2025

**EMbase**

First subject heading:internet addiction

'Addiction Disorder, Internet':ab,ti or 'Addiction Disorders, Internet':ab,ti or 'Disorder, Internet Addiction':ab,ti or 'Disorders, Internet Addiction':ab,ti or 'Internet Addiction Disorders':ab,ti or 'Internet Addiction':ab,ti or 'Addiction, Internet':ab,ti or 'Addictions, Internet':ab,ti or 'Internet Addictions':ab,ti or 'Internet Gaming Disorder':ab,ti or 'Disorder, Internet Gaming':ab,ti or 'Disorders, Internet Gaming':ab,ti or 'Gaming Disorder, Internet':ab,ti or 'Internet Gaming Disorders':ab,ti or 'Gaming Disorders, Internet':ab,ti or 'Smartphone Addiction':ab,ti or 'Addiction, Smartphone':ab,ti or 'Addictions, Smartphone':ab,ti or 'Smartphone Addictions':ab,ti or 'Social Media Addiction':ab,ti or 'Addiction, Social Media':ab,ti or 'Addictions, Social Media':ab,ti or 'Media Addiction, Social':ab,ti or 'Media Addictions, Social':ab,ti or 'Social Media Addictions':ab,ti

Second subject heading: Psychosocial Intervention

'Intervention, Psychosocial':ab,ti or 'Interventions, Psychosocial':ab,ti or 'Psychosocial Interventions':ab,ti or 'Psychological Intervention':ab,ti or 'Intervention, Psychological':ab,ti or 'Interventions, Psychological':ab,ti or 'Psychological Interventions':ab,ti

Third subject heading:'randomized controlled trial':ab,ti or 'randomized':ab,ti or 'placebo':ab,ti

**Cochrane**

First subject heading:Internet Addiction Disorder

(Addiction Disorder, Internet):ti,ab,kw OR (Addiction Disorders, Internet):ti,ab,kw OR (Disorder, Internet Addiction):ti,ab,kw OR (Disorders, Internet Addiction):ti,ab,kw OR (Internet Addiction Disorders):ti,ab,kw OR (Internet Addiction):ti,ab,kw OR (Addiction, Internet):ti,ab,kw OR (Addictions, Internet):ti,ab,kw OR (Internet Addictions):ti,ab,kw OR (Internet Gaming Disorder):ti,ab,kw OR (Disorder, Internet Gaming):ti,ab,kw OR (Disorders, Internet Gaming):ti,ab,kw OR (Gaming Disorder, Internet):ti,ab,kw OR (Gaming Disorders, Internet):ti,ab,kw OR (Internet Gaming Disorders):ti,ab,kw OR (Smartphone Addiction):ti,ab,kw OR (Addiction, Smartphone):ti,ab,kw OR (Addictions, Smartphone):ti,ab,kw OR (Smartphone Addictions):ti,ab,kw OR (Social Media Addiction):ti,ab,kw OR (Addiction, Social Media):ti,ab,kw OR (Addictions, Social Media):ti,ab,kw OR (Media Addiction, Social):ti,ab,kw OR (Media Addictions, Social):ti,ab,kw OR (Social Media Addictions):ti,ab,kw

Second subject heading:Psychosocial Intervention

(Intervention, Psychosocial):ti,ab,kw OR (Interventions, Psychosocial):ti,ab,kw OR (Psychosocial Interventions):ti,ab,kw OR (Psychological Intervention):ti,ab,kw OR (Intervention, Psychological):ti,ab,kw OR (Interventions, Psychological):ti,ab,kw OR (Psychological Interventions):ti,ab,kw

**Search syntax for Chinese-language databases：**

The following search syntax is presented in Chinese.

**CNKI：**

Search syntax A：

（主题：游戏成瘾）OR（主题：游戏障碍）OR（主题：网络游戏成瘾）OR（主题：网络游戏障碍）AND（主题：心理干预）OR（主题：心理治疗）AND（摘要：随机对照(精确)）OR（摘要：RCT(精确)）

Search syntax B：

（主题：游戏成瘾）OR（主题：游戏障碍）OR（主题：网络游戏成瘾）OR（主题：网络游戏障碍）AND（主题：心理治疗）OR（主题：心理干预）AND（摘要：RCT(精确)）OR（摘要：随机对照(精确)）

**Wanfang：**

Search syntax A：

（主题：游戏成瘾）OR（主题：游戏障碍）OR（主题：网络游戏成瘾）OR（主题：网络游戏障碍）AND（主题：心理干预）OR（主题：心理治疗）AND（摘要：随机对照(精确)）OR（摘要：RCT(精确)）

Search syntax B：

（主题：游戏成瘾）OR（主题：游戏障碍）OR（主题：网络游戏成瘾）OR（主题：网络游戏障碍）AND（主题：心理治疗）OR（主题：心理干预）AND（摘要：RCT(精确)）OR（摘要：随机对照(精确)）

**VIP：**

[(((((题名或关键词=游戏成瘾 OR 题名或关键词=游戏障碍) OR 题名或关键词=网络游戏成瘾) OR 题名或关键词=网络游戏障碍) AND (任意字段=心理治疗 OR 任意字段=心理干预)) AND 任意字段=随机)](http://qikan.cqvip.com/Qikan/search/index?LngMySearHistoryIdGuid=92a198ca-4ddd-4c08-ba40-1a094302a76e&from=Qikan_Article_History)
